# Topical Management Strategies for Acneiform Eruptions Induced by Cancer Drugs

**DOI:** 10.7759/cureus.86447

**Published:** 2025-06-20

**Authors:** Maria Aftab, Syeda Sakina, Sania Khan, Hifza Ishtiaq, Maryam Atta, Asma Atta, Sheharyar Khalid Rahim

**Affiliations:** 1 Geriatrics, Royal Infirmary of Edinburgh, Edinburgh, GBR; 2 Medicine, Dr. Sakina Clinics, Wah, PAK; 3 Oncology, Shaukat Khanum Memorial Cancer Hospital and Research Centre, Lahore, PAK; 4 Medicine, Abbas Institute of Medical Sciences, Muzaffarabad, PAK; 5 Anesthesia, Azad Jammu Kashmir Medical College, Muzaffarabad, PAK; 6 Internal Medicine, DHQ Teaching Hospital, Kohat, PAK

**Keywords:** acneiform eruptions, cancer therapies, corticosteroids, topical treatments, tyrosine kinase inhibitors

## Abstract

Background: Acneiform eruptions are common dermatological side effects of cancer therapies, particularly those involving tyrosine kinase inhibitors (TKIs) and epidermal growth factor receptor (EGFR) inhibitors, and can significantly impact patients' quality of life.

Objective: The primary objective of this study was to evaluate the effectiveness of various topical therapies for managing cancer drug-induced acneiform eruptions and to assess patient-reported outcomes. Secondary objectives include comparing tolerability profiles and examining associations between malignancy types, treatment regimens, and eruption patterns.

Materials and methods: This prospective observational study was conducted from January to December 2023 at the Departments of Dermatology, Abbas Institute of Medical Sciences (AIMS), Muzaffarabad, and Pakistan Institute of Medical Sciences (PIMS), Islamabad, Pakistan. A total of 116 patients aged 18 years or older who developed acneiform eruptions following cancer therapy were enrolled. Participants received topical treatments, including corticosteroids, benzoyl peroxide, metronidazole, retinoids, and antibiotics (clindamycin or erythromycin). Clinical assessments included lesion count, severity grading, and patient-reported outcomes (pain, itching, quality of life), recorded at baseline and at 2-, 4-, and 8-week follow-ups. Data analysis was performed using IBM SPSS Statistics for Windows, Version 26.0 (Released 2018; IBM Corp., Armonk, NY, US). Treatment efficacy comparisons were made using chi-square tests for categorical variables, t-tests for continuous variables, and multiple linear regression to identify independent predictors of lesion reduction.

Results: Antibiotics were the most commonly used treatment (30 patients, 25.86%), followed by benzoyl peroxide (27 patients, 23.28%) and corticosteroids (25 patients, 21.55%). By week 8, 99 patients (85.35%) presented with only mild lesions. Topical antibiotics (clindamycin/erythromycin) demonstrated the highest effectiveness, with a mean lesion count reduction of 7.2 ± 1.8 and severity reduction score of 3.6 ± 1.2, achieving an overall efficacy of 54%. Multiple linear regression analysis identified a later onset of acneiform eruptions (>5 weeks after therapy initiation) as a significant predictor of greater lesion reduction (β = -0.30, p = 0.047). Metronidazole was associated with the lowest incidence of adverse effects (2 patients, 13.33%). Both topical antibiotics and benzoyl peroxide significantly reduced lesion count and severity.

Conclusion: These findings suggest that topical antibiotics, benzoyl peroxide, and corticosteroids are effective in managing cancer therapy-induced acneiform eruptions, with notable improvements in clinical severity and patient-reported outcomes. Additionally, a later onset of eruptions may be associated with a more favorable therapeutic response.

## Introduction

Advances in immunomodulatory agents and personalized cancer therapies have significantly improved survival outcomes for many cancer patients [1]. Among the most common and disruptive dermatological side effects associated with these advances are acneiform outbreaks [2]. Often linked to tyrosine kinase inhibitors (TKIs) and epidermal growth factor receptor (EGFR) inhibitors, these skin reactions are more than mere cosmetic concerns; they can cause significant discomfort, reduce quality of life, and even compromise treatment compliance [3,4].

Unlike classic acne vulgaris, acneiform eruptions commonly affect the face, chest, and upper back and appear as papulopustular rashes without comedones [5]. They usually emerge within the first few weeks of therapy and often coincide with peak drug activity [6]. While their presence may indicate treatment efficacy, particularly with EGFR-targeted therapies [7], if left unmanaged, these eruptions may lead to dose modifications or discontinuation of therapy. Therefore, effective, evidence-based therapeutic approaches that address both clinical and psychological impacts, integrating dermatologic care with oncologic needs, are essential [8].

Due to their favorable safety profiles, limited systemic activity, and minimal absorption, topical therapies have gained popularity in managing acneiform eruptions [9]. Commonly used agents include benzoyl peroxide, metronidazole, retinoids, corticosteroids, and antibiotics (such as erythromycin and clindamycin), administered either alone or in combination [5,10]. These treatments exert their effects through mechanisms such as restoration of follicular keratinization, as well as antibacterial and anti-inflammatory actions [11].

Despite widespread clinical use, comparative data on the efficacy of these treatments remain limited, and current management practices vary considerably. Furthermore, differences in pathophysiological mechanisms raise questions about the appropriateness of extrapolating treatments from acne vulgaris to drug-induced acneiform eruptions. As newer oncologic therapies become more prevalent, these dermatologic adverse effects are likely to increase. This underscores the need to evaluate and optimize topical treatment strategies that can effectively control acneiform eruptions without interfering with cancer therapy.

The primary objective of this study is to evaluate the effectiveness of topical therapies, including clindamycin/erythromycin, benzoyl peroxide, metronidazole, retinoids, and corticosteroids, in managing acneiform eruptions induced by cancer treatments, with a focus on lesion reduction, symptom relief, and improvement in patient-reported outcomes. Secondary objectives include comparing the tolerability and adverse effect profiles of these topical agents and investigating associations between cancer type and stage, specific oncologic treatment regimens, and eruption patterns, to help identify high-risk subgroups and predict therapy-related cutaneous side effects.

## Materials and methods

Ethical considerations

This prospective observational study was conducted at two tertiary care centers in Pakistan. Ethical approval was obtained from the Institutional Review Board (IRB) of Abbas Institute of Medical Sciences, Muzaffarabad (Ref. No. 6234/AIMS/2024). Eligible patients who developed acneiform eruptions during cancer therapy and consented to participate were enrolled using convenience sampling. Written informed consent was obtained from all participants prior to inclusion, including agreement for the collection and use of clinical data and photographs, where applicable. Participants were assured of the confidentiality of their identity and personal information, and any photographs used for documentation or research dissemination were anonymized to prevent identification. All study procedures were conducted in accordance with institutional guidelines and national ethical standards.

Study design and setting

This was a prospective, observational cohort study conducted at the Department of Dermatology, Abbas Institute of Medical Sciences (Muzaffarabad) and Pakistan Institute of Medical Sciences (Islamabad), Pakistan. The study was carried out over a period of 12 months, from January to December 2023. Patients were recruited from both outpatient and inpatient services. The dermatology department collaborated closely with the oncology unit to facilitate patient referrals and ensure consistent follow-up assessments.

Inclusion and exclusion criteria

Patients were eligible for inclusion if they were 18 years or older, of either gender, and had developed acneiform eruptions following the initiation of cancer therapy, specifically involving EGFR inhibitors, TKIs, or immune checkpoint inhibitors (immunotherapy). All participants were required to provide written informed consent and demonstrate a willingness to comply with scheduled follow-up visits.

Exclusion criteria included current use of systemic medications for acneiform eruptions at the time of enrollment, prior discontinuation of cancer therapy for reasons unrelated to dermatological side effects, a known history of moderate-to-severe acne vulgaris prior to cancer treatment, and the presence of other dermatologic conditions, such as rosacea, folliculitis, or seborrheic dermatitis, that could interfere with accurate assessment of acneiform eruptions.

Sample size and sampling technique

A total of 116 eligible patients were enrolled using a non-probability convenience sampling method. The sample size was determined based on the anticipated patient volume during the study period, resource availability, and logistical feasibility within the department. This number was deemed sufficient to identify relevant trends and patterns aligned with the study objectives.

Data collection and clinical assessment

Upon enrollment, participants underwent a comprehensive dermatological examination, and relevant information was recorded using a structured case report form (Supplemental material). Collected data included demographic variables such as age, gender, body mass index (BMI), and the presence of comorbidities. Cancer-specific details were also documented, including the malignancy type and stage, the specific oncologic treatment regimen, and dosage information.

The most commonly observed malignancies were non-small cell lung cancer (NSCLC) and colorectal cancer, with the majority of patients presenting at stage III or IV. Predominant oncologic therapies included EGFR inhibitors (e.g., gefitinib, erlotinib at 250-300 mg daily), TKIs (e.g., imatinib, sorafenib), and immune checkpoint inhibitors. Dosage and duration of these therapies were recorded to assess their association with the onset and severity of acneiform eruptions.

Acneiform eruption data included time of onset relative to cancer treatment, lesion morphology, and anatomical distribution. Severity was graded according to the National Cancer Institute's Common Terminology Criteria for Adverse Events (NCI-CTCAE) version 5.0 [12]: Grade 1 (mild), papules/pustules covering <10% of body surface area (BSA) without associated symptoms; Grade 2 (moderate), 10%-30% BSA with symptoms such as itching or tenderness; and Grade 3 (severe), >30% BSA or involvement leading to psychosocial impact or functional limitation. To minimize observer variability and enhance reliability, two independent dermatologists performed lesion counts (papules, pustules, nodules).

Treatment and follow-up

Patients received individualized topical treatment plans tailored to lesion severity, distribution, and standard dermatologic guidelines. Therapeutic agents included corticosteroids, clindamycin, benzoyl peroxide, metronidazole, and retinoids. Appropriate formulations and vehicles were selected to optimize tolerability and efficacy: creams or gels were applied to localized areas (e.g., the face), while body washes or solutions (e.g., clindamycin or benzoyl peroxide washes) were for more extensive involvement of the trunk or back, enhancing tolerability and efficacy across different body regions.

Participants were followed at 2, 4, and 8 weeks following the initiation of therapy. At each visit, clinical evaluations included lesion count, severity grading, and documentation of adverse effects. Patient-reported outcomes, such as pain, itching, and burning, were assessed using the Visual Analog Scale (VAS), while quality of life was measured using the Dermatology Life Quality Index (DLQI) [13] at each time point.

Statistical analysis

Data were analyzed using IBM SPSS Statistics for Windows, Version 26.0 (Released 2018; IBM Corp., Armonk, NY, US). Descriptive statistics were used to summarize demographic and clinical characteristics: categorical variables were reported as frequencies and percentages, while continuous variables were expressed as means ± standard deviations. Chi-square tests were applied to compare categorical variables. Differences in mean lesion count and severity reductions across topical treatment groups were assessed using one-way analysis of variance (ANOVA). A p < 0.05 was considered statistically significant. When ANOVA results were significant, post hoc pairwise comparisons were performed using Tukey’s Honestly Significant Difference (HSD) test to identify specific group differences. Multivariate linear regression analyses were conducted to determine independent predictors of lesion count reduction and severity improvement, adjusting for relevant demographic and clinical covariates.

## Results

The demographic and clinical characteristics of the participants are summarized in Table 1. The majority were female patients (61, 52.59%) and between 31 and 45 years old (42, 36.21%). Most patients received EGFR inhibitors (44, 37.93%), followed by TKIs (38, 32.76%) and immunotherapy (34, 29.31%). Acneiform eruptions occurred within the first two weeks of therapy in 68 patients (58.62%). Chi-square analysis revealed no statistically significant differences across age groups (χ² = 3.5, p = 0.32), gender (χ² = 0.12, p = 0.73), type of cancer treatment (χ² = 2.8, p = 0.25), or timing of acneiform eruption onset (χ² = 4.1, p = 0.13), indicating a balanced distribution of these characteristics among study participants.

**Table 1:** Demographic and clinical characteristics of the study participants The low chi-square (χ²) values indicate no statistically significant associations among the demographic variables, as expected in baseline characteristics. All p-values were greater than 0.05, consistent with a balanced or randomly distributed sample across groups.

Characteristic	Category	Number of patients (n; %)	χ²	p-value
Age group	18-30 years	28 (24.14%)	3.5	0.32
	31-45 years	42 (36.21%)		
	46-60 years	30 (25.86%)		
	61+ years	16 (13.79%)		
Gender	Male	55 (47.41%)	0.12	0.73
	Female	61 (52.59%)		
Type of cancer treatment	Epidermal growth factor receptor (EGFR) inhibitors	44 (37.93%)	2.8	0.25
	Tyrosine kinase inhibitors (TKIs)	38 (32.76%)		
	Immunotherapy	34 (29.31%)		
Onset of acneiform eruptions	0-2 weeks	68 (58.62%)	4.1	0.13
	3-5 weeks	39 (33.62%)		
	>5 weeks	9 (7.76%)		

**Figure 1:**
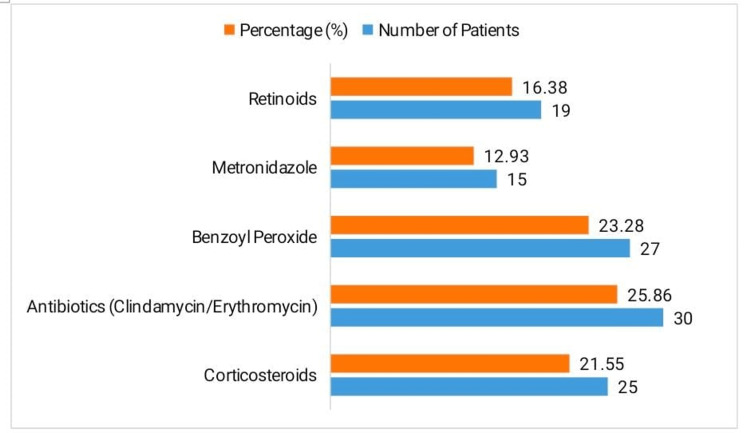
Topical treatment modalities used in acneiform eruptions

The most frequently used topical treatments were antibiotics (clindamycin/erythromycin) in 30 patients (25.86%), followed by benzoyl peroxide (27 patients, 23.28%), corticosteroids (25 patients, 21.55%), retinoids (19 patients, 16.38%), and metronidazole (15 patients, 12.93%), as illustrated in Figure 1.

Over the 8-week follow-up period, there was a marked improvement in severity of acneiform eruptions, with a significant reduction in moderate and severe cases (Table 2). By week 8, 99 patients (85.35%) presented with mild eruptions, compared to 72 patients (62.07%) at baseline.

**Table 2:** Severity of acneiform eruptions at baseline and at 2-, 4-, and 8-week follow-ups Severity grading based on the National Cancer Institute Common Terminology Criteria for Adverse Events version 5.0 (NCI-CTCAE v5.0): Grade 1 (mild), papules/pustules covering <10% body surface area (BSA), with or without symptoms such as pruritus or tenderness; Grade 2 (moderate), papules/pustules covering 10%-30% BSA with symptoms impacting instrumental activities of daily living; and Grade 3 (severe), papules/pustules covering >30% BSA with severe symptoms limiting self-care activities of daily living.

Severity level	Baseline (n; %)	Two weeks (n; %)	Four weeks (n; %)	Eight weeks (n; %)
Grade 1 (mild)	72 (62.07)	84 (72.41)	92 (79.32)	99 (85.35)
Grade 2 (moderate)	35 (30.17)	25 (21.56)	18 (15.51)	12 (10.34)
Grade 3 (severe)	9 (7.76)	7 (6.03)	6 (5.17)	5 (4.31)

Patient-reported outcomes for pain, itching, and quality of life are summarized in Table 3. By week 8, the mean itching score decreased significantly from 7.1 ± 1.5 at baseline to 2.1 ± 0.8. Corresponding improvements in pain and quality of life scores were also observed.

**Table 3:** Patient-reported outcomes (itching, pain, and quality of life impact) at baseline and at 2-, 4-, and 8-week follow-ups

Symptom	Baseline	Two weeks	Four weeks	Eight weeks
Itching (scale: 0-10)	7.1 ± 1.5	5.2 ± 1.2	3.5 ± 1.0	2.1 ± 0.8
Pain (scale: 0-10)	6.5 ± 1.8	4.8 ± 1.3	3.1 ± 1.0	1.9 ± 0.7
Quality of life impact (scale: 0-10)	7.4 ± 2.0	5.6 ± 1.5	3.8 ± 1.2	2.5 ± 1.0

The efficacy of various topical therapies in reducing lesion count and severity is presented in Table 4. The greatest mean reduction in lesion count was observed with antibiotics (clindamycin/erythromycin) at 7.2 ± 1.8, followed by benzoyl peroxide (6.5 ± 2.0) and corticosteroids (6.3 ± 2.1). In terms of overall treatment effectiveness, antibiotics demonstrated the highest efficacy (54%, 30 patients), followed by benzoyl peroxide (49%, 27 patients) and corticosteroids (47%, 25 patients).

**Table 4:** Effectiveness of topical treatments in reducing lesion count and severity

Topical treatment	Lesion count reduction (mean ± SD)	Severity reduction (mean ± SD)	Overall effectiveness (%)
Corticosteroids	6.3 ± 2.1	3.1 ± 1.3	47.00%
Antibiotics (clindamycin/erythromycin)	7.2 ± 1.8	3.6 ± 1.2	54.00%
Benzoyl peroxide	6.5 ± 2.0	3.3 ± 1.4	49.00%
Metronidazole	5.6 ± 1.9	2.9 ± 1.0	42.50%
Retinoids	6.0 ± 2.3	3.0 ± 1.1	45.00%

In this study, all topical treatments demonstrated varying degrees of effectiveness in reducing lesion count and severity of cancer therapy-induced acneiform eruptions. Antibiotics (clindamycin/erythromycin) yielded the greatest mean reductions in lesion count (7.2 ± 1.8) and severity (3.6 ± 1.2), with statistically significant improvements (F = 3.78, p = 0.011). Benzoyl peroxide and corticosteroids also showed significant efficacy, with lesion count reductions of 6.5 ± 2.0 and 6.3 ± 2.1 and severity reductions of 3.3 ± 1.4 (F = 3.2, p = 0.032) and 3.1 ± 1.3 (F = 2.67, p = 0.042), respectively. Retinoids demonstrated moderate effectiveness (lesion count reduction: 6.0 ± 2.3; severity reduction: 3.0 ± 1.1; F = 2.44, p = 0.045), whereas metronidazole showed the least improvement and did not reach statistical significance (lesion count reduction: 5.6 ± 1.9; severity reduction: 2.9 ± 1.0; F = 2.01, p = 0.059). Adverse effects were most commonly reported with benzoyl peroxide (6 patients, 22.22%), followed by corticosteroids (5 patients, 20.00%), retinoids (3 patients, 15.79%), antibiotics (4 patients, 13.33%), and metronidazole (2 patients, 13.33%). These findings (Table 5) support the preferential use of topical antibiotics and benzoyl peroxide for managing acneiform eruptions, balancing both clinical efficacy and tolerability.

**Table 5:** Adverse effects and ANOVA summary for topical treatments *Statistical significance was set at p < 0.05. ANOVA tests were used to compare mean lesion and severity reductions among treatment groups. Reported adverse effects reflect established tolerability profiles for each agent.

Topical treatment	Adverse effects reported	Patients affected (%)	Lesion count reduction (mean ± SD)	Severity reduction (mean ± SD)	ANOVA F-value	ANOVA p-value
Corticosteroids	Skin thinning, irritation	5 (20.00%)	6.3 ± 2.1	3.1 ± 1.3	2.67	0.042*
Antibiotics (clindamycin/erythromycin)	Dryness, redness	4 (13.33%)	7.2 ± 1.8	3.6 ± 1.2	3.78	0.011*
Benzoyl peroxide	Burning, skin irritation	6 (22.22%)	6.5 ± 2.0	3.3 ± 1.4	3.2	0.032*
Metronidazole	Irritation, dryness	2 (13.33%)	5.6 ± 1.9	2.9 ± 1.0	2.01	0.059
Retinoids	Peeling, irritation	3 (15.79%)	6.0 ± 2.3	3.0 ± 1.1	2.44	0.045*

One-way ANOVA revealed statistically significant differences in lesion and severity reductions among the treatment groups (p < 0.05). Post hoc Tukey’s tests (Table 6) confirmed that antibiotics were significantly more effective than both metronidazole and retinoids. Although benzoyl peroxide also demonstrated strong efficacy, it was associated with the highest incidence of adverse effects. These findings support the use of antibiotics and benzoyl peroxide as primary topical treatments for managing cancer therapy-induced acneiform eruptions, while emphasizing the importance of balancing clinical effectiveness with tolerability.

**Table 6:** Post hoc pairwise comparisons (Tukey’s HSD) of the effectiveness of different topical treatments in managing acneiform eruptions *p-values indicate statistically significant differences (p < 0.05) in treatment efficacy between groups. HSD: honestly significant difference.

Comparison	Mean difference	p-value
Antibiotics vs. metronidazole	1.6	0.020*
Antibiotics vs. retinoids	1.2	0.030*
Benzoyl peroxide vs. metronidazole	0.9	0.058
Corticosteroids vs. metronidazole	0.7	0.072
Retinoids vs. metronidazole	0.4	0.133

Multiple linear regression analysis (Table 7) revealed that the timing of acneiform eruption onset was a significant predictor of lesion count reduction. Patients who developed eruptions after five weeks of initiating cancer therapy showed a greater reduction in lesion count (β = -0.30, p = 0.047). No significant associations were observed for age group, gender, or type of cancer treatment, suggesting these variables did not independently affect treatment efficacy in lesion reduction.

**Table 7:** Multiple linear regression analysis for predictors of lesion count reduction *p-values indicate statistical significance (p < 0.05). EGFR: epidermal growth factor receptor.

Predictor variable		Beta coefficient	Standard error (SE)	t-value	p-value
Age group (reference: 18-30 years)	31-45 years	0.15	0.1	1.5	0.14
	46-60 years	-0.05	0.11	-0.45	0.65
	61+ years	-0.2	0.13	-1.54	0.13
Gender (reference: male)		0.1	0.08	1.25	0.21
Cancer treatment (reference: EGFR inhibitors)	Tyrosine kinase inhibitors	0.05	0.09	0.56	0.58
	Immunotherapy	-0.12	0.1	-1.2	0.23
Onset of acneiform eruptions (weeks; reference: 0-2 weeks)	3-5 weeks	-0.18	0.11	-1.64	0.11
	>5 weeks	-0.3	0.15	-2	0.047

## Discussion

Acneiform eruptions are common dermatological side effects of cancer therapies, particularly TKIs and EGFR inhibitors, and can significantly impair patients’ quality of life. This study aimed to evaluate the effectiveness, safety, and patient tolerability of various topical treatments in managing these eruptions, with a focus on symptom relief, lesion resolution, and patient-reported outcomes. Our findings provide valuable insights into the comparative efficacy of several topical agents for cancer therapy-induced acneiform eruptions.

The demographic profile of our cohort aligned well with existing literature. Most patients (36.21%) were between 31 and 45 years old, with a slight female predominance (52.59%) [14]. Consistent with previous studies, over half (58.62%) of acneiform eruptions appeared within the first two weeks of initiating cancer therapy, underscoring the importance of early recognition and intervention to prevent treatment disruption [2].

The effectiveness of topical treatments, including benzoyl peroxide, corticosteroids, metronidazole, retinoids, and antibiotics (clindamycin/erythromycin), in managing acneiform eruptions was evaluated. In this study, antibiotics (clindamycin/erythromycin) were the most effective, with a mean lesion count reduction of 7.2 ± 1.8 and severity reduction of 3.6 ± 1.2. These findings align with previous research that supports antibiotics as a first-line therapy due to their anti-inflammatory and antibacterial properties [15]. Benzoyl peroxide, commonly used for acne vulgaris, also demonstrated notable efficacy (lesion count reduction of 6.5 ± 2.0 and severity reduction of 3.3 ± 1.4), supporting its broader application in managing drug-induced acneiform eruptions [16].

Topical therapies demonstrated statistically significant improvements over time, with antibiotics and benzoyl peroxide showing the greatest reductions in lesion count and severity (p = 0.011 and p = 0.032, respectively), and corticosteroids also achieving significant efficacy (p = 0.042). The improvements were progressive across the 2-, 4-, and 8-week follow-ups, reinforcing the reliability of these treatments in managing acneiform eruptions.

Corticosteroids, despite their anti-inflammatory potency, were slightly less effective than antibiotics and benzoyl peroxide, achieving a lesion count reduction of 6.3 ± 2.1 and a severity reduction of 3.1 ± 1.3. This finding aligns with previous studies indicating that while corticosteroids help reduce inflammation, their effectiveness in managing acneiform eruptions may be limited due to potential side effects such as skin thinning [17]. Retinoids exhibited the least efficacy in our acute setting, with a lesion count reduction of 6.0 ± 2.3 and severity reduction of 3.0 ± 1.1. This may be attributed to their benefits being more evident with prolonged use in chronic acne rather than in the context of acute, drug-induced eruptions [18].

Patient-reported outcomes further supported the clinical findings: itching, pain, and quality of life improved significantly over the 8-week follow-up. Notably, itching scores decreased from 7.1 ± 1.5 at baseline to 2.1 ± 0.8 at week 8, consistent with previous reports demonstrating symptom relief following topical treatment [19]. Pain and overall quality of life also showed marked improvement, highlighting the positive impact of effective dermatologic management on patient comfort.

Adverse effects were consistent with known profiles: benzoyl peroxide had the highest rate of skin irritation (22.22%), manifesting as burning and dryness, in line with prior studies [20]. Corticosteroids caused skin thinning and irritation in 20% of patients, echoing concerns about their long-term safety in acneiform eruptions [21].

In terms of oncologic context, the majority of patients with acneiform eruptions had NSCLC or colorectal cancer, primarily at advanced stages (III and IV), reflecting the need for targeted therapies in these populations. EGFR inhibitors such as gefitinib and erlotinib were commonly used at standard doses (250-300 mg daily), while TKIs like imatinib and sorafenib were also frequently administered. Acneiform eruptions typically developed early, within the first two weeks of treatment, especially among those on higher-intensity regimens. These observations reinforce the well-established link between targeted cancer therapies and early dermatologic adverse effects, emphasizing the need for vigilant monitoring and tailored management strategies.

Study strengths and limitations

This study has several strengths. The prospective design, relatively large sample size (116 patients), and the structured evaluation of multiple topical therapies provide a valuable foundation for understanding the dermatologic management of acneiform eruptions induced by targeted cancer treatments. The inclusion of both objective (lesion count, severity grading) and subjective (pain, itching, and quality of life) outcome measures enhances clinical relevance. Additionally, conducting the study across two centers improves the generalizability of findings compared to a single-center design. Stratifying results by drug class and malignancy type offers insight into which patient subgroups may be at higher risk of developing cutaneous adverse effects.

Certain limitations should be acknowledged. Although the multicenter approach enhances representativeness, the use of convenience sampling could introduce selection bias, potentially affecting the diversity of the study population. Reliance on self-reported symptoms (itching and pain) may introduce response bias despite the use of validated tools. Furthermore, while topical corticosteroids were included as treatment options, their application (especially over large body areas such as the trunk) may exacerbate acneiform eruptions and cause local or systemic side effects. Future randomized multicenter trials with standardized treatment protocols and consideration of appropriate vehicles for wider application (e.g., body washes) are recommended to validate and expand upon these findings.

## Conclusions

According to the findings of this study, the most effective treatments for cancer therapy-induced acneiform eruptions were topical antibiotics (clindamycin/erythromycin), benzoyl peroxide, and corticosteroids. These therapies significantly reduced lesion severity and alleviated patient-reported symptoms such as pain and itching, thereby enhancing patients’ overall quality of life. Despite certain limitations, the results support the clinical utility of topical treatment approaches for managing this common dermatologic side effect. These findings also highlight the potential for improved patient comfort and adherence to cancer therapy. Further research is warranted to optimize treatment protocols and assess long-term outcomes.

## Appendices

Supplemental material 

**Table 8:** Questionnaire for participant data collection

Questionnaire for participant data collection	Response/notes
Participant ID:	
Age (years):	
Gender:	☐ Male ☐ Female ☐ Other
Type of cancer treatment:	☐ EGFR inhibitors ☐ Tyrosine kinase inhibitors ☐ Immunotherapy ☐ Other: _______
Type of malignancy:	
Stage of cancer:	☐ I ☐ II ☐ III ☐ IV
Time from start of treatment to acneiform eruption (weeks):	
Severity of acneiform eruption at baseline:	☐ Mild (Grade 1) ☐ Moderate (Grade 2) ☐ Severe (Grade 3)
Topical treatment used:	☐ Antibiotics (clindamycin/erythromycin) ☐ Benzoyl peroxide ☐ Corticosteroids ☐ Retinoids ☐ Metronidazole ☐ Other: _______
Lesion count at baseline:	
Lesion count at 2 weeks:	
Lesion count at 4 weeks:	
Lesion count at 8 weeks:	
Severity at 2 weeks:	☐ Mild ☐ Moderate ☐ Severe
Severity at 4 weeks:	☐ Mild ☐ Moderate ☐ Severe
Severity at 8 weeks:	☐ Mild ☐ Moderate ☐ Severe
Itching score (0-10) at baseline:	
Itching score (0-10) at 2 weeks:	
Itching score (0-10) at 4 weeks:	
Itching score (0-10) at 8 weeks:	
Pain score (0-10) at baseline:	
Pain score (0-10) at 2 weeks:	
Pain score (0-10) at 4 weeks:	
Pain score (0-10) at 8 weeks:	
Quality of life impact score (0-10) at baseline:	
Quality of life impact score (0-10) at 2 weeks:	
Quality of life impact score (0-10) at 4 weeks:	
Quality of life impact score (0-10) at 8 weeks:	
Adverse effects experienced:	☐ Burning ☐ Irritation ☐ Skin thinning ☐ Dryness ☐ Redness ☐ Peeling ☐ Other: _______
Additional comments/notes:	
